# Uterine fibroids increase the risk of thyroid goiter and thyroid nodules

**DOI:** 10.1038/s41598-022-10625-x

**Published:** 2022-04-22

**Authors:** Jin-Sung Yuk, Jung Min Kim

**Affiliations:** 1 Department of Obstetrics and Gynecology, Sanggye Paik Hospital, Inje University College of Medicine, Seoul, Republic of Korea; 2 Department of Internal Medicine, Sanggye Paik Hospital, Inje University College of Medicine, Seoul, Republic of Korea

**Keywords:** Thyroid diseases, Endocrine reproductive disorders

## Abstract

Uterine fibroid and benign thyroid disease are both common diseases in women. This study aimed to evaluate whether these diseases are related. We established the uterine fibroid group according to diagnosis and surgery codes using the Korea National Health Insurance data from 2009 to 2020. All women from 20 to 50 years old who underwent uterine myomectomy from 2009 to 2020 were identified. We selected the control group by performing a 1:1 propensity score matching on age at 5-year intervals, socioeconomic status (SES), region, Charlson comorbidity index (CCI), menopause, and year among women who visited a medical institution for a health check-up. Thyroid disease cases were selected using the thyroid disease diagnosis code and thyroid-associated laboratory tests. A total of 181,419 patients were included in the uterine fibroid and control groups. The median age of each group was 40 (range, 35 ~ 44) and 40 (range, 35 ~ 45) years old, respectively. Benign thyroid disease affected 1162 (0.6%) in the uterine fibroid group and 1137 (0.6%) in the control group. Among the benign thyroid diseases, hypothyroidism was the most common in both groups, followed by a nontoxic single thyroid nodule. The uterine fibroid group had a higher risk of thyroid goiter (hazard ratio (HR) 1.169, 95% confidence interval (CI) 1.022–1.338), nontoxic single thyroid nodule (HR 1.268, 95% CI 1.182–1.361), and total thyroid disease (HR 1.078, 95% CI 1.036–1.121) in stratified Cox regression analysis adjusted for age, SES, region, CCI, parity, menopause, hypertension, diabetes, dyslipidemia, systemic lupus erythematosus, irritable bowel syndrome, Crohn’s disease, and endometriosis than the control group. The results suggest that women with uterine fibroids have an increased risk of thyroid goiters and thyroid nodules. Although the mechanism is not well known, estrogen and iodide might be a link between uterine fibroids and thyroid goiters and nodules. Future studies that prospectively follow women with uterine fibroids across a lifetime are needed.

## Introduction

Uterine fibroid, also called myoma, is the most common gynecological benign tumor in premenopausal women^1^. It consists mostly of monoclonal cells from the myometrium, the smooth muscle layer of the uterus, fibroblasts, and the extracellular matrix. Uterine fibroids occur more frequently in black women, women with an early menarche, women using hormonal contraceptives prior to 16 years of age, and those with a high body mass index (BMI). Uterine fibroids express more estrogen receptors and progesterone receptors than normal myometrium. Ovarian steroids, estradiol, and progesterone promote the growth of uterine fibroids.

Thyroid nodules are more common in women than in men^2^. Estrogen affects thyroid function, regulates thyroid-stimulating hormone (TSH), and may have a role in the formation of thyroid nodules^3^. Thyroid dysfunction is more common in women than men. This appears to be due to sex differences in immune function, as in many autoimmune diseases. More than 80% of patients with thyroiditis and its resulting hypothyroidism have antithyroid antibodies^4^. According to US data, thyroid autoantibodies against thyroid peroxidase are found in 3% of teenage boys, 7% of teenage girls, 12% of men over 80 years of age, and 30% of women^5^.

Little is known about the association between uterine fibroid and thyroid disease. In a study conducted in 1989, women who had undergone a hysterectomy for uterine fibroids had significantly more frequent pathological thyrotrophin-releasing hormone (TRH)/TSH stimulation test results and more antiperoxidase antibody and/or thyroglobulin antibodies than the control group^6^. Recent studies have shown that women with fibroids have a higher risk of thyroid cancer^7^ and thyroid nodules^8,9^. An association between fibroids and overt hypothyroidism has been reported^10^. However, these studies have limitations, such as a study with a small number of patients^8,9^, a study not performing propensity matching despite the availability of large-scale national data^7^, and not performing a comprehensive study of benign thyroid disease^7–10^.

Using a nationwide population-based database for a cohort study can provide significantly more data than a cohort based on multiple institutions. Therefore, this study aimed to evaluate the relationship between uterine fibroids and benign thyroid disease using Korean Health Insurance Review and Assessment Service (HIRA) data.

## Materials and methods

### Database

i. Korea provides national health insurance services to all Koreans (approximately 51 million people) by law^11^. Therefore, Korea's National Health Insurance Corporation provides medical information for most diseases (diagnosis code, operation code, laboratory examination code, prescription drug information, type of medical insurance, region, hospital), except for exceptional cases such as cosmetic surgery. Medical institutions in Korea make a claim to the Korea Health Insurance Corporation for medical insurance costs. Since there may be disputes over medical expenses between medical institutions and the Korea Health Insurance Corporation, the agency that mediates them is the HIRA. Therefore, the HIRA has access to most of the national health insurance information in Korea. HIRA data are publicly available. HIRA data can be requested from the HIRA data site (http://opendata.hira.or.kr).
ii. This retrospective cohort study used health insurance data provided by the HIRA from January 1, 2009, to December 31, 2020.

### Selection of participants

i. In this study, Korea Health Insurance Medical Care Expenses (2016, 2019 version) were used to select subjects and outcomes for surgery and examination codes. The International Classification of Diseases, 10th revision (ICD-10) for diagnosis codes was used.
ii. This study selected the uterine fibroid group, 20 ~ 50 year old women, with the code for diagnosing uterine fibroids (D25.x) and the code for myomectomy (R4121 ~ 9). In addition, to increase the accuracy of the experimental group, only women who visited medical institutions more than 2 times for uterine fibroids were included.
iii. As a control group, women aged 20 ~ 50 years who visited a medical institution for a routine health check-up were extracted. Among these women, if there was a diagnosis code (D25.x) of uterine fibroids even once, they were excluded from the study.
iv. Those who had any cancer (any C.xx) or visited a medical institution more than once with a diagnosis code for thyroid disease (E00 ~ 07) 180 days before the selection date were excluded. In addition, cases that had undergone myomectomy before January 01, 2011, or visited the hospital for a health check-up were excluded for wash out.
v. For the subjects thus extracted, 1:1 propensity score matching was performed on age at 5-year intervals, socioeconomic status (SES), region of residence, Charlson comorbidity index (CCI), menopause, and year.

### Outcome

i. A patient with thyroid disease is defined as visiting medical institutions 3 times with thyroid diagnosis codes and undergoing thyroid-related tests (thyroid scan, triiodothyronine (T3), free T3, T3 uptake, reverse T3, thyroxine (T4), free T4, thyroglobulin, TSH, antiperoxidase antibody, antithyroglobulin antibody, thyroid function test, thyroid-stimulating immunoglobulin, T4-binding globulin, thyroid sonography).
ii. Thyroid diseases are divided into the hypothyroid group (E03.4, E03.5, E03.8, E03.9), hyperthyroidism (E05.0, E05.1, E05.2, E05.5, E05.8, E05.9), autoimmune thyroid group (E06.2, E06.3, E06.5, E06.9), goiter group (E04.0, E04.2, E04.8, E04.9), nodular group (E04.1) and others (E07.8, E07.9).

### Variables

i. The independent variables were age at surgery, SES, and region. Ages were categorized into 5-year intervals. When the type of medical insurance was medical aid, which corresponds to Medicaid in the United States, it was defined as low SES. If the location of the medical institution was not metropolitan, it was defined as a rural region. Parity was defined as a delivery performed if there was a diagnostic code or technical code (primipara or multipara) related to a birth. Menopause, hypertension, diabetes mellitus, and dyslipidemia were defined as having the corresponding diagnosis when a medical institution was visited twice or more with the corresponding diagnosis code. Systemic lupus erythematosus (SLE), irritable bowel syndrome (IBD), Crohn’s disease, and endometriosis were defined as having the corresponding diagnosis when a medical institution was visited 3 or more times with the corresponding diagnosis code.
ii. Comorbidity was calculated using the diagnosis code from 1 year before the date of participation to the date of participation in the study, using the method of Quan et al. to calculate the CCI^12^.

### Statistics

i. All statistics mainly used SAS Enterprise Guide 6.1 (SAS Institute Inc.). As a supplement, R 3.0.2 (The R Foundation for Statistical Computing) was used.
ii. A two-sided test was performed for all statistics in this study. Statistical significance was defined when the p value was less than or equal to 0.05.
iii. In this study, the paired t-test (parametric) and Wilcoxon signed-rank test (nonparametric) were used to analyze continuous variables, and the Cochran–Mantel–Haenszel test was used to analyze categorical variables.
iv. In this study, stratified Cox regression analysis was performed for the adjustment of various confounding factors.
v. Stratified Cox regression analysis was performed only for patients who had undergone uterine fibroid removal by laparotomy as a case group to confirm the robustness of the results of this study.
vi. When the missing value was less than 10%, the listwise deletion method was performed, and when the missing value was more than 10%, the regression imputation method was performed.

### Ethics

i. This study was approved by the Institutional Review Board of Inje University Sanggye Paik Hospital (No.: SGPAIK 2021-02-005). As these data are public and nonpersonally identifiable, the Institutional Review Board of Inje University Sanggye Paik Hospital IRB waived informed consent.
ii. The data used in this study were provided after removing variables that could identify individuals in the HIRA database. In addition, data analysis in this study can only be performed on HIRA's server, and the raw data cannot be exported. Therefore, since it is impossible to specify any individual included in the data, there is no possible harm to the individual included in the data. This study does not disadvantage the involved individuals because the data do not contain personally identifiable information. Therefore, this study does not require informed consent from subjects according to the Bioethics and Safety Act of South Korea. This study was conducted in accordance with the guidelines of South Korea’s Bioethics and Safety Act.
iii. Although this study used data provided by the HIRA, the HIRA and the Korean Ministry of Health and Welfare have no financial or other conflicts of interest in this study.

## Results

### Clinical characteristics (Fig. 1, Table 1)

**Figure 1:**
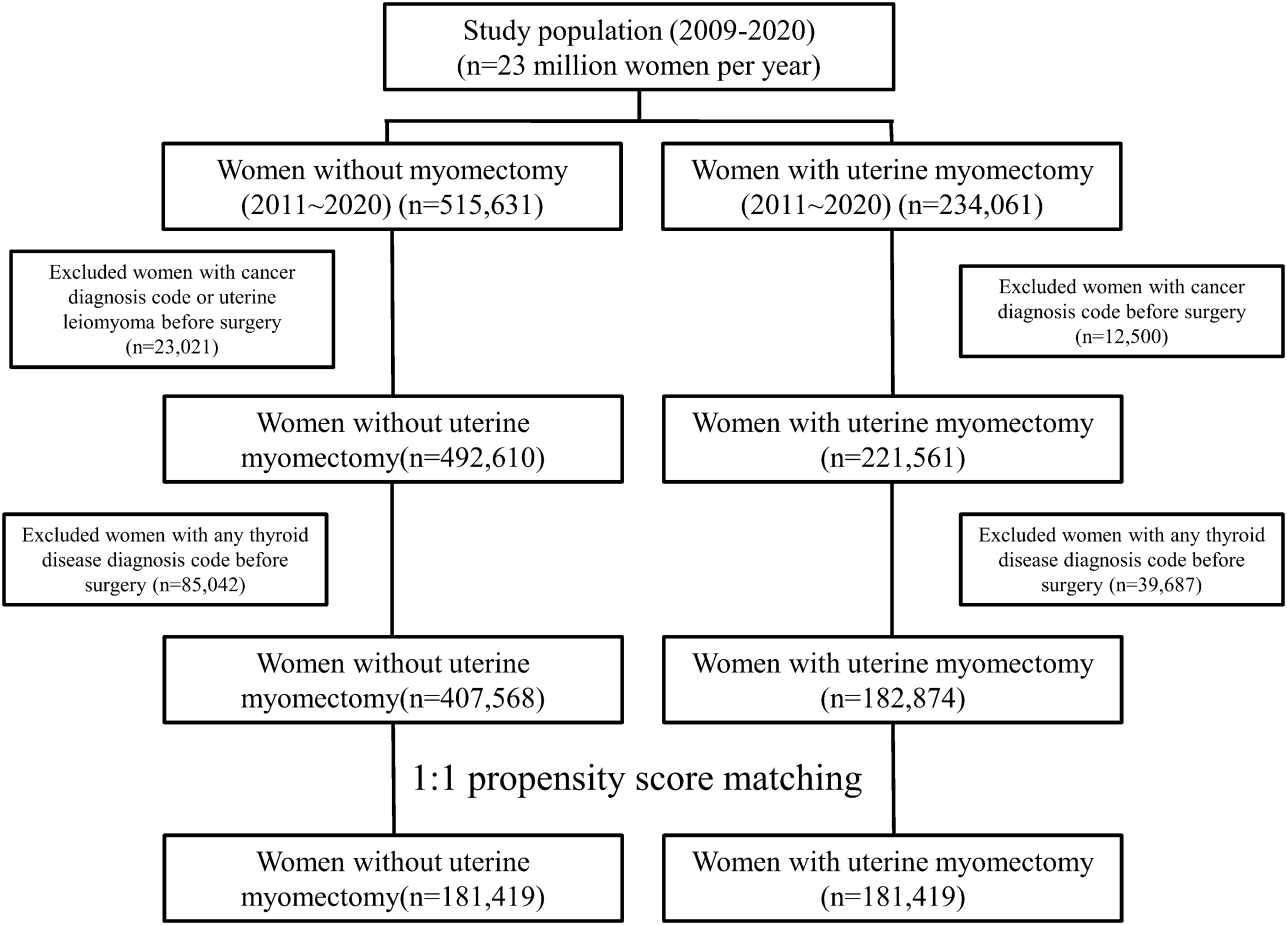
Flowchart for selecting case and control groups in this study using HIRA data. *HIRA* The Health Insurance Review and Assessment Service.

**Table 1:** Characteristics of women with uterine fibroid and the control group from the HIRA claim data.

	Control	Uterine fibroid	Total	P value	Standardized difference
Number of participants	181,419	181,419	362,838		
Median age (years)	40 [35–45]	40 [35–44]	40 [35–44]	< 0.001	− 0.033
**Age at inclusion (years)**				< 0.001	0.109
20–24	2016 (1.1)	2004 (1.1)	4020 (1.1)		
25–29	12,456 (6.9)	12,440 (6.9)	24,896 (6.9)		
30–34	30,841 (17)	30,775 (17)	61,616 (17)		
35–39	39,134 (21.6)	42,535 (23.4)	81,669 (22.5)		
40–44	48,270 (26.6)	52,926 (29.2)	101,196 (27.9)		
45–49	48,702 (26.8)	40,739 (22.5)	89,441 (24.7)		
**SES**				0.708	− 0.001
Mid ~ high SES	179,497 (98.9)	179,520 (99)	359,017 (98.9)		
Low SES	1922 (1.1)	1899 (1)	3821 (1.1)		
**Region**				< 0.001	− 0.097
Urban area	103,771 (57.2)	112,361 (61.9)	216,132 (59.6)		
Rural area	77,648 (42.8)	69,058 (38.1)	146,706 (40.4)		
**CCI**				0.400	0.011
0	145,277 (80.1)	145,741 (80.3)	291,018 (80.2)		
1	23,150 (12.8)	22,518 (12.4)	45,668 (12.6)		
≥ 2	12,992 (7.2)	13,160 (7.3)	26,152 (7.2)		
**Parity in cohort**				0.097	0.009
0	161,834 (89.2)	162,263 (89.4)	324,097 (89.3)		
1	12,763 (7)	12,350 (6.8)	25,113 (6.9)		
≥ 2	6822 (3.8)	6806 (3.8)	13,628 (3.8)		
**Menopause**				< 0.001	− 0.130
No	178,441 (98.4)	178,738 (98.5)	357,179 (98.4)		
Yes	2978 (1.6)	2681 (1.5)	5659 (1.6)		
**Hypertension**				< 0.001	
No	169,400 (93.4)	170,607 (94)	340,007 (93.7)		
Yes	12,019 (6.6)	10,812 (6)	22,831 (6.3)		
**DM**				< 0.001	
No	172,727 (95.2)	174,169 (96)	346,896 (95.6)		
Yes	8692 (4.8)	7250 (4)	15,942 (4.4)		
**Dyslipidemia**				< 0.001	
No	156,805 (86.4)	157,623 (86.9)	314,428 (86.7)		
Yes	24,614 (13.6)	23,796 (13.1)	48,410 (13.3)		
**SLE**				< 0.001	
No	181,049 (99.8)	181,180 (99.9)	362,229 (99.8)		
Yes	370 (0.2)	239 (0.1)	609 (0.2)		
**IBD**				< 0.001	
No	158,459 (87.3)	161,086 (88.8)	319,545 (88.1)		
Yes	22,960 (12.7)	20,333 (11.2)	43,293 (11.9)		
**Crohn's disease**				< 0.001	
No	181,333 (100)	181,383 (100)	362,716 (100)		
Yes	86 (0)	36 (0)	122 (0)		
**Endometriosis**				< 0.001	
No	177,511 (97.8)	158,076 (87.1)	335,587 (92.5)		
Yes	3908 (2.2)	23,343 (12.9)	27,251 (7.5)		

*CCI* Charlson comorbidity index, *DM* diabetes mellitus, *IBD* irritable bowel syndrome, *SES* socioeconomic status, *SLE* systemic lupus erythematosus.

Data are expressed as the number (%) or median [25 percentile, 75 percentile].

In this study, 181,419 patients were extracted for the uterine fibroid and control groups (Fig. 1). The median age of each group was 40 (range, 35 ~ 44) and 40 (range, 35 ~ 45) years, respectively. The standardized differences of the variables subjected to propensity score matching were all less than or equal to 0.2 in absolute value. The detailed characteristics of the patients are shown in Table 1.

### Thyroid disease in women in the uterine fibroid group or control group (Table 2)

**Table 2:** The cases of thyroid disease among women with uterine fibroid or controls in the HIRA claim data (1:1 matching).

	Control	Uterine fibroid	Total	P value
Number of women	181,419	181,419	362,838	
**Hypothyroidism**				0.015
No	179,224 (98.8)	179,061 (98.7)	358,285 (98.7)	
Yes	2195 (1.2)	2358 (1.3)	4553 (1.3)	
**Hyperthyroidism**				0.611
No	180,379 (99.4)	180,402 (99.4)	360,781 (99.4)	
Yes	1040 (0.6)	1017 (0.6)	2057 (0.6)	
**Autoimmune thyroid disease**				0.554
No	180,534 (99.5)	180,509 (99.5)	361,043 (99.5)	
Yes	885 (0.5)	910 (0.5)	1795 (0.5)	
**Goiter**				0.006
No	180,958 (99.7)	180,871 (99.7)	361,829 (99.7)	
Yes	461 (0.3)	548 (0.3)	1009 (0.3)	
**Nontoxic single thyroid nodule**				< 0.001
No	179,792 (99.1)	179,281 (98.8)	359,073 (99)	
Yes	1627 (0.9)	2138 (1.2)	3765 (1)	
**Other thyroid disease**				0.6
No	180,282 (99.4)	180,257 (99.4)	360,539 (99.4)	
Yes	1137 (0.6)	1162 (0.6)	2299 (0.6)	
**Total thyroid disease**				< 0.001
Absent	175,672 (96.8)	175,071 (96.5)	350,743 (96.7)	
Present	5747 (3.2)	6348 (3.5)	12,095 (3.3)	

HIRA, Health Insurance Review & Assessment Service.

Data are expressed as the number (%).

Benign thyroid disease affected 1162 (0.6%) in the uterine fibroid group and 1137 (0.6%) in the control group (Table 2). Among the benign thyroid diseases in both groups, hypothyroidism was the most common, followed by a nontoxic single thyroid nodule.

### Stratified Cox regression analysis of thyroid disease in women with uterine fibroids (Fig. 2, Table 3)

**Figure 2:**
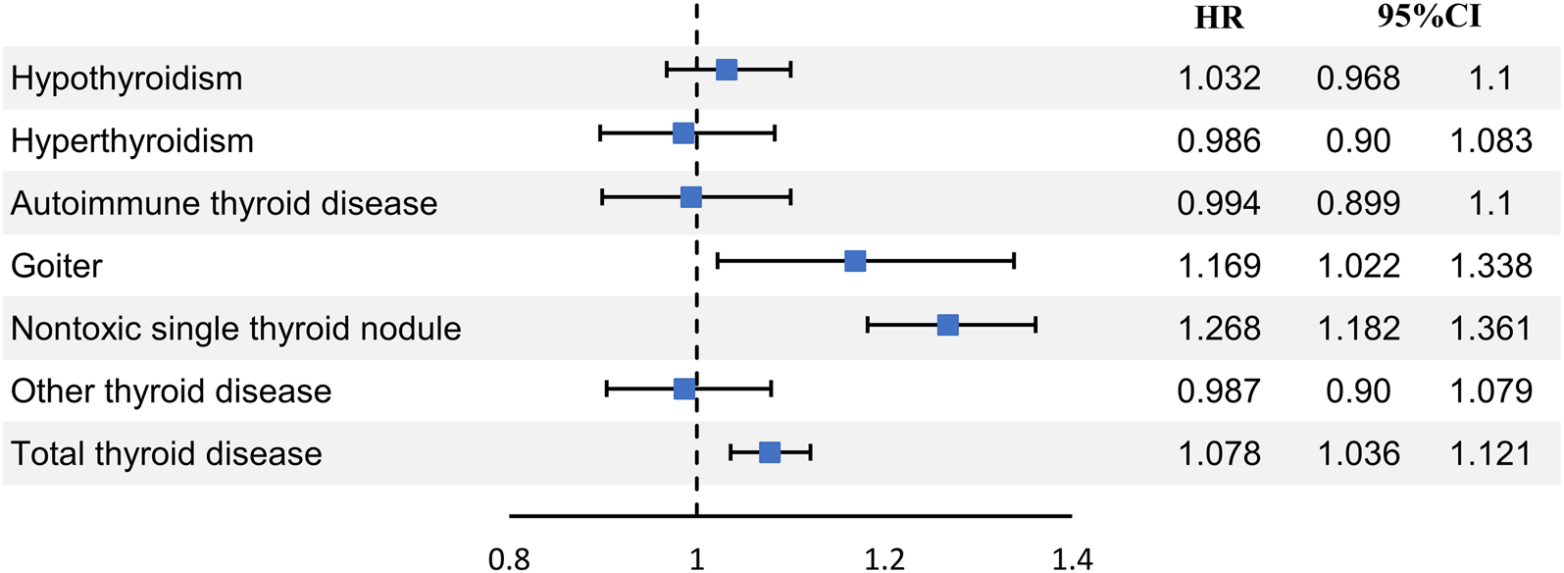
Conditional logistic regression analysis of thyroid disease in women with uterine fibroids from the HIRA data (after matching).

**Table 3:** Stratified Cox regression analysis of thyroid disease in patients with uterine fibroid from the HIRA data (after matching).

	Unadjusted		Adjusted Model 1^a^		Adjusted Model 2^b^	
	HR (95% CI)	P value	HR (95% CI)	P value	HR (95% CI)	P value
Hypothyroidism	1.05 (0.989–1.114)	0.109	1.039 (0.978–1.104)	0.218	1.032 (0.968–1.1)	0.333
Hyperthyroidism	0.97 (0.888–1.06)	0.5	0.985 (0.9–1.078)	0.735	0.986 (0.897–1.083)	0.761
Autoimmune thyroid disease	1.015 (0.924–1.115)	0.756	1 (0.909–1.101)	0.993	0.994 (0.899–1.1)	0.914
Goiter	1.177 (1.037–1.335)	0.011	1.152 (1.013–1.312)	0.032	1.169 (1.022–1.338)	0.023
Nontoxic single thyroid nodule	1.292 (1.21–1.379)	< 0.001	1.275 (1.192–1.365)	< 0.001	1.268 (1.182–1.361)	< 0.001
Other thyroid disease	1.006 (0.926–1.093)	0.882	1.005 (0.923–1.094)	0.908	0.987 (0.904–1.079)	0.779
Total thyroid disease	1.089 (1.05–1.13)	< 0.001	1.082 (1.042–1.124)	< 0.001	1.078 (1.036–1.121)	< 0.001

*CCI* Charlson comorbidity index, *CI* confidence interval, *DM* diabetes mellitus, *HIRA* health insurance review & assessment service, *HR* hazard ratio, *IBD* irritable bowel syndrome, *SES* socioeconomic status, *SLE* systemic lupus erythematosus.

^a^Each thyroid disease ~ Age per 5 years + Low SES + region + CCI + parity + menopause.

^b^Each thyroid disease ~ Age per 5 years + Low SES + region + CCI + parity + menopause + hypertension + DM + dyslipidemia + SLE + IBD + Crohn's disease + endometriosis.

In stratified Cox regression analysis adjusted for age, SES, region, CCI, parity, menopause, hypertension, DM, dyslipidemia, SLE, IBD, Crohn’s disease, endometriosis, etc., the uterine fibroid group had a significantly higher risk of thyroid goiter (hazard ratio (HR) 1.169, 95% confidence interval (CI) 1.022–1.338), nontoxic single thyroid nodule (HR 1.268, 95% CI 1.182–1.361), and total thyroid disease (HR 1.078, 95% CI 1.036–1.121) (Fig. 2). On the other hand, there was no difference in the risk of hypothyroidism (HR 1.032, 95% CI 0.968–1.1), hyperthyroidism (HR 0.986, 95% CI 0.897–1.083), autoimmune thyroid disease (HR 0.994, 95% CI 0.899–1.1), or other thyroid diseases (HR 0.987, 95% CI 0.904–1.079).

### Incidence of total thyroid disease in women in the uterine fibroid group or control group (Table 4)

**Table 4:** Case/person-years of total thyroid disease in participants with or without myomectomy.

	Control	Uterine fibroid
Total	5747/854,561 (673)	6348/869,229 (730)
**Age at inclusion (years)**		
20–24	52/9459 (550)	41/9475 (433)
25–29	520/60,793 (855)	490/61,102 (802)
30–34	1243/153,679 (809)	1401/153,366 (914)
35–39	1189/184,315 (645)	1510/206,626 (731)
40–44	1284/227,096 (565)	1634/255,192 (640)
45–49	1459/219,218 (666)	1272/183,538 (693)
**SES**		
Mid ~ high SES	5669/844,652 (671)	6280/859,536 (731)
Low SES	78/9908 (787)	68/9763 (697)
**Region**		
Urban area	3599/490,098 (734)	4247/544,976 (779)
Rural area	2148/364,462 (589)	2101/324,323 (648)
**CCI**		
0	4441/681,673 (651)	4889/694,658 (704)
1	790/110,896 (712)	920/109,381 (841)
≥ 2	516/61,991 (832)	539/65,260 (826)
**Parity in cohort**		
0	5417/788,525 (687)	6039/803,932 (751)
1	245/45,299 (541)	245/44,175 (555)
≥ 2	85/20,737 (410)	64/21,192 (302)
**Menopause**		
No	5598/838,846 (667)	6219/855,923 (727)
Yes	149/15,714 (948)	129/13,376 (964)
**Hypertension**		
No	5329/801,336 (665)	5953/820,936 (725)
Yes	418/53,225 (785)	395/48,364 (817)
**DM**		
No	5483/818,755 (670)	6086/838,440 (726)
Yes	264/35,806 (737)	262/30,859 (849)
**Dyslipidemia**		
No	4977/758,814 (656)	5615/775,042 (724)
Yes	770/95,746 (804)	733/94,257 (778)
**SLE**		
No	5733/853,139 (672)	6342/868,312 (730)
Yes	14/1,421 (985)	6/987 (608)
**IBD**		
No	4827/750,724 (643)	5459/778,192 (701)
Yes	920/103,837 (886)	889/91,107 (976)
**Crohn's disease**		
No	5742/854,220 (672)	6348/869,162 (730)
Yes	5/341 (1466)	0/137 (0)
**Endometriosis**		
No	5600/838,995 (667)	5504/764,009 (720)
Yes	147/15,566 (944)	844/105,290 (802)

*CCI* Charlson comorbidity index, *DM* diabetes mellitus, *IBD* irritable bowel syndrome, *SES* socioeconomic status, *SLE* systemic lupus erythematosus.

Data are expressed as the case/person-years (case/100,000 person-years).

The incidence of total thyroid disease according to the person-years was 730 per 100,000 person-years in the uterine fibroid group and 673 per 100,000 person-years in the control group. The detailed rates according to each variable are shown in Table 4.

### Sensitivity test

In a sensitivity test analyzed using only patients with laparotomy, other thyroid diseases increased (HR 1.257, 95% CI 1.163–1.359), and goiter tended to increase (HR 1.151, 95% CI 0.99–1.338). There were no differences for the other diseases.

## Discussion

### Principal findings

This study evaluated whether uterine fibroids are associated with benign thyroid diseases, such as hypothyroidism, autoimmune thyroiditis, thyroid goiter, thyroid nodule, or hyperthyroidism. Stratified Cox regression analysis showed that uterine fibroids were significantly associated with thyroid goiter, thyroid nodule, and total thyroid disease but not with hypothyroidism, hyperthyroidism or autoimmune thyroid disease.

### Results

Previous studies have evaluated the association between uterine fibroids and thyroid diseases^7–10^. Patients with uterine fibroids have significantly higher rates of thyroid nodules^8,9^. In an analysis using the national health insurance research database of Taiwan, similar to this study, Sun et al. reported that uterine fibroids increased the risk of thyroid cancer^7^. Our findings are consistent with previous studies showing that patients with uterine fibroids have higher rates of benign thyroid diseases, such as thyroid goiter and thyroid nodules.

### Clinical implications

Why is the incidence of thyroid goiter and thyroid nodules high in patients with uterine fibroids? What are the common causative factors of uterine fibroids and thyroid goiter and thyroid nodules?

First, female hormones are a common cause of both uterine fibroids and thyroid goiter and nodules.

Uterine fibroids are mainly comprised of an extracellular matrix, have a low mitotic index and usually grow slowly^13^. Uterine fibroids are more common in premenopausal women, and their risk factors include early menarche, the use of hormonal contraceptives before the age of 16, and a high BMI^1^. Estradiol and progesterone promote the growth and size of uterine fibroids^14^. Uterine fibroids are more responsive to female hormones than normal myometrium, probably because uterine fibroids express more estrogen receptors (ERs) and progesterone receptors than normal myometrium^15^. As such, uterine fibroids could be estrogen-dependent diseases.

What about benign thyroid disease? Thyroid disease is also more prevalent between puberty and menopause^16^. Thyroid nodules or thyroid cancer are three times more common in women^16,17^. The growth of benign thyroid nodules slows after menopause^18^. Women who experience menopause over 55 years of age have more thyroid nodules than women who experience menopause under 50 years of age. Women with 40 or more reproductive years have a higher risk of thyroid nodules than women with less than 35 reproductive years^19^. These epidemiological data suggest an effect of estrogen on thyroid disease. Factors estimated to reflect high levels of estrogen exposure, such as uterine fibroids and reproductive years, were found to increase the risk of thyroid cancer^20^.

Estrogen promotes cell growth in primary cultures of human thyrocytes from benign and malignant thyroid nodules and of thyroid cancer cell lines^21,22^. Estrogen enhances the metastatic properties of thyroid cells, including adhesion, migration, and invasiveness, and promotes proliferation^23^. Estrogen promotes growth through both ERα and ERβ in thyroid cells. Nevertheless, in thyroid cancer cells, ERα is increased, promoting tumorigenesis, and ERβ is decreased, thus acting as a tumor suppressor^22^. ERα is also increased in uterine fibroids^24^. Estrogen interacts with ER in immune cells and alters apoptotic pathways, such as Bcl-2 family protein activity and nuclear factor kappa B activity^22,25,26^.

In summary, estrogen can be seen as a cause of thyroid nodules, including thyroid cancer, but the causal relationship and mechanism are unclear.

Second, iodine may be a factor in the association between uterine fibroids and thyroid goiters^10^. Iodine is a trace element and is reduced to iodide and then absorbed in the stomach and duodenum. Iodide is taken up into tissues through sodium iodide symporter (NIS) in the thyroid gland, ovary, uterine endometrium, stomach, and breast^27^. NIS is important for thyroid iodine uptake and thyroid hormone synthesis^28^. Estrogen reduces NIS gene expression^29^ and reduces iodide uptake^30^, so insufficient iodine intake may be a factor in the development of goiter and hypothyroidism.

It is well known that iodine deficiency causes goiter. Iodine deficiency also increases estrogen activity. Iodine deficiency decreases cytochrome P4501A1 and 1B1. This results in a decrease in estrone and estradiol metabolism. In addition, iodine deficiency causes estrogen-induced transcription by reducing the activity of BRCA1, an inhibitor of ERα transcription^31^. Therefore, iodine deficiency may cause an increase in estrogen, which can promote the development of both thyroid goiter and uterine fibroids. However, since Korea is not an iodine-deficient country^32^, this explanation does not seem reasonable. Additionally, the prevalence of goiter shows no sex difference in iodine-sufficient regions^33^. However, this part is controversial because there is no information on iodine intake, such as urine iodine amount, in the target group of this study.

### Research implications

Future studies that prospectively follow women with uterine fibroids across a lifetime would overcome the limitations of our analysis.

### Strengths and limitations

This study has some limitations. First, there can be inaccuracy in the diagnosis. A diagnosis code does not mean a disease. Diagnosis codes are likely to be inaccurate, as they are collected to reimburse health care services. As mentioned before, it is likely that autoimmune thyroiditis was only labelled as diagnosis code ‘hypothyroidism.’ Second, HIRA data does not include specific information about the patient. We could not detect or correct for changes caused by external interference other than diseases, such as drugs. We could not correct for BMI, family history, etc. Third, this study is limited to a treatment group that has undergone myomectomy, so there is a limitation in that it cannot represent all patients with uterine fibroids.

However, this study has strengths because this study analyzed a large number of patients (more than 180,000). As the HIRA data are from a large-scale national health insurance database, this study is representative of the Korean population. The patient samples are comprehensive but also contain specific information, such as prescribed medications. Because the patient samples passed the validity test, they are effective for estimating the effect on the entire population. Additionally, surveillance bias is unlikely to exist in this study. Compared to the control health examination group, patients who had undergone myomectomy for uterine fibroids tend not to visit the hospital or undergo additional examinations.

### Conclusions

In conclusion, uterine fibroids could be associated with thyroid goiters and nodules. However, uterine fibroids were not associated with hypothyroidism, hyperthyroidism, or autoimmune thyroid disease. Although the mechanism is not well known, estrogen and iodide might be a link between uterine fibroids and thyroid goiters and nodules. Future studies that prospectively follow women with uterine fibroids across a lifetime are needed.
